# Time for What? Dissociating Explicit Timing Tasks through Electrophysiological Signatures

**DOI:** 10.1523/ENEURO.0351-23.2023

**Published:** 2024-02-21

**Authors:** Fernanda D. Bueno, Anna C. Nobre, André M. Cravo

**Affiliations:** ^1^Center for Mathematics, Computing and Cognition (CMCC), Federal University of ABC (UFABC), São Bernardo do Campo 09606-045, Brazil; ^2^Oxford Centre for Human Brain Activity, Wellcome Centre for Integrative Neuroimaging, University of Oxford, Oxford OX3 7JX, United Kingdom; ^3^Department of Experimental Psychology, University of Oxford, Oxford OX2 6GG, United Kingdom

**Keywords:** EEG, perception, timing

## Abstract

Estimating durations between hundreds of milliseconds and seconds is essential for several daily tasks. Explicit timing tasks, which require participants to estimate durations to make a comparison (time for perception) or to reproduce them (time for action), are often used to investigate psychological and neural timing mechanisms. Recent studies have proposed that mechanisms may depend on specific task requirements. In this study, we conducted electroencephalogram (EEG) recordings on human participants as they estimated intervals in different task contexts to investigate the extent to which timing mechanisms depend on the nature of the task. We compared the neural processing of identical visual reference stimuli in two different tasks, in which stimulus durations were either perceptually compared or motorically reproduced in separate experimental blocks. Using multivariate pattern analyses, we could successfully decode the duration and the task of reference stimuli. We found evidence for both overlapping timing mechanisms across tasks as well as recruitment of task-dependent processes for comparing intervals for different purposes. Our findings suggest both core and specialized timing functions are recruited to support explicit timing tasks.

## Significance Statement

Understanding the underlying psychological and neural mechanisms involved in timing is essential for advancing our knowledge. This study aimed to investigate the influence of task context on timing mechanisms by conducting electroencephalogram (EEG) recordings on human participants. Employing multivariate pattern analyses, we compared neural processing during two distinct timing tasks: perceptual comparison and motor reproduction of intervals. Our results revealed a combination of shared timing mechanisms across tasks and the engagement of task-specific processes, highlighting the coexistence of core and specialized timing functions in explicit timing tasks. These findings contribute to a deeper understanding of how timing mechanisms operate and adapt to different task demands.

## Introduction

Interval timing, the ability to estimate durations in the hundreds of milliseconds to seconds, is essential for daily tasks. Although this ability is commonly treated as a single skill, recent studies have proposed that how we process temporal durations may depend on how we use this information (Lewis and Miall, 2003; Coull and Nobre, 2008; Breska and Ivry, 2016; van Wassenhove et al., 2019).

One organizing principle of timing tasks is how temporal information will be used and measured (Coull and Nobre, 2008; Shalev et al., 2019). A major distinction is between implicit and explicit timing tasks (Coull and Nobre, 2008). Implicit timing tasks do not require participants to report temporal durations; however, time impacts performance on another task-relevant factor. For example, temporal cues can facilitate sensory and motor performance (Rohenkohl et al., 2012, 2014; Nobre and van Ede, 2018). In explicit timing tasks, temporal durations are the main objective, and participants report them in some fashion. Convergent evidence from lesion and correlational studies suggests that different neural systems contribute to implicit versus explicit timing functions (Coull and Nobre, 2008; Breska and Ivry, 2016).

Although explicit timing tasks have traditionally been treated as a homogeneous category, how participants provide explicit reports can differ significantly across tasks. For example, in motor tasks, participants estimate durations to execute timed actions, while in perceptual timing tasks, they estimate durations to assess or compare them with a reference. If explicit timing relies on a shared common mechanism that serves various types of explicit reports, then temporal encoding should unfold similarly regardless of the motor or sensory demands of the task. In contrast, differences in temporal encoding as a function of task demands would suggest heterogeneous and highly specialized local temporal encoding mechanisms. Identifying differences in temporal encoding could also have broader implications for interpreting temporal processing differences in explicit versus implicit tasks since the demands often differ in these two classes of tasks. Explicit timing tasks often rely on perceptual timing judgments, whereas implicit timing tasks rely on speeded motor actions. The possibility remains that differences attributed to explicit versus implicit timing may, at least partly, reflect differences in reporting demands instead. Understanding whether the perceptual and motor demands of tasks lead to neural differences would thus help qualify interpretations regarding the factors determining neural processing of timing.

Previous studies exploring perceptual and motor temporal judgments have primarily relied on behavioral (Keele et al., 1985; Ivry and Hazeltine, 1995; Merchant et al., 2008a,b; Rammsayer and Troche, 2014) and fMRI measures (Bueti et al., 2008; Wiener et al., 2010; Nani et al., 2019; Naghibi et al., 2023). Both methods suggest a combination of general and goal-directed processes involved in perceptual and sensorimotor timing. However, while behavioral findings can offer insight into how we express our time estimations, they do not let us compare whether or how different temporal processing stages are affected by task requirements. Similarly, functional brain imaging can identify common and distinct brain areas activated depending on the task goals but without indicating whether the dynamical patterns of activations within and across regions are comparable.

Electrophysiological studies in humans using EEG can offer valuable additional insights into how intervals are encoded and how task demands can influence different temporal processing stages. Many studies have investigated event-related potentials and timing (for a review, see Kononowicz et al., 2018), most focusing on a single task or comparing the activity between temporal and nontemporal tasks. Recent developments in multivariate pattern analysis (MVPA) have demonstrated that EEG data also contain rich spatial information that can be used to decode neural states (Stokes et al., 2015). These methods have started to be applied to temporal tasks, revealing how task goals and context can influence how time is encoded in brain activity (Bueno and Cravo, 2021; Damsma et al., 2021).

This study investigated whether multivariate analysis of time-resolved EEG signals can distinguish between explicit timing of durations in tasks stressing perception versus action. We designed an experiment in which participants viewed a reference interval in each trial and, in different blocks, had to reproduce the duration or compare it to a probe. The visual stimulus and set of durations/intervals were identical between conditions, enabling us to explore how EEG signals during and after the reference interval were modulated by the stimulus duration, task, and interaction. With this approach, we aimed to advance the understanding of the neural mechanisms underlying perceptual and motor timing and how they differ and overlap.

## Materials and Methods

### Data availability

All materials resulting from this study are openly available. Please see (https://osf.io/cr6yg/) to access the task, analysis code, and raw and preprocessed data.

### Participants

The experimental protocol was approved by The Research Ethics Committee of the Federal University of ABC (UFABC), where the study took place. Study implementation followed approved guidelines and regulations (CAEE: 03607118.4.0000.5594).

Thirty-three human volunteers participated in the study after giving informed consent. Data from a final sample of 29 volunteers were fully analyzed (mean age, 23 years; age range of 18–35; 14 females). Data from the four additional participants were excluded from the analysis. The reasons included the following: loss of data due to an energy blackout during the experiment; loss of data due to computer memory issues during data collection; computer memory issues and excessive noise during data collection; and excessive data loss (19%) during artifact removal (see below). All participants had normal or corrected vision and declared being free from psychological or neurological diseases.

### Stimuli and procedures

**Figure 1.**
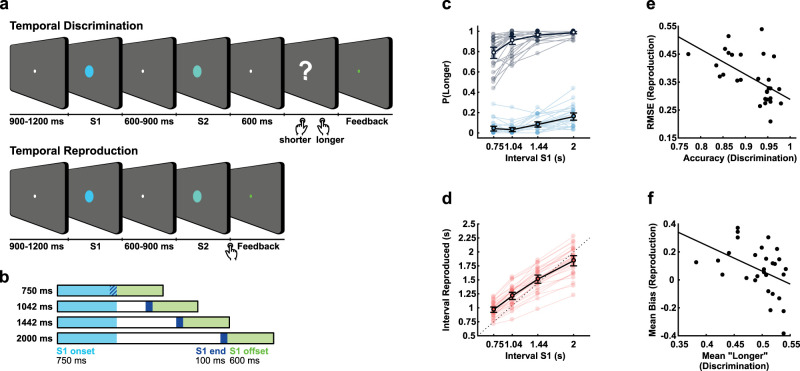
Experimental design and behavioral results. ***a***, Schematic illustration of the discrimination and reproduction tasks. ***b***, Schematic illustration of the three segments used in the analysis, all relative to S1. The light blue area represents the period from 0 to 750 ms from S1 onset, whereas the dark blue area represents the final 100 ms before offset for all four sample intervals. The green area depicts the period analyzed after S1 offset. ***c***, Behavioral results for the discrimination task. The proportion of “longer than S1” responses according to S1 interval (750, 1,042, 1,442, or 2,000 ms) and whether S2 was shorter (light blue) or longer (gray) than S1. ***d***, Behavioral results for the reproduction task depict the mean of reproduced durations for each S1 interval. For ***c*** and ***d***, the light-colored circles depict each participant's values, whereas black circles depict mean results for all participants. Error bars represent the standard error of the mean. ***e***, Correlation between the accuracy of responses (discrimination) and RMSE (reproduction; robust skipped Spearman correlation; *r* = −0.60; 95% CI between −0.79 and −0.29; *p* < 0.001). ***f***, Correlation between the mean of “longer” (discrimination) and mean bias (difference between reproduced and reference interval; *r* = −0.42; 95% CI between −0.66 and −0.09; *p* = 0.02). In ***e*** and ***f***, circles depict individual values and the line represents the correlation between values.

The experiment consisted of two computerized tasks presented in different blocks, temporal discrimination and temporal reproduction (Fig. 1*a*), combined with EEG recordings. The stimuli were presented using Psychtoolbox (Brainard, 1997) v.3.0 package in Octave on a VIEWPixx monitor with a vertical refresh rate of 120 Hz, placed at ∼52 cm from the participant. Responses were collected via a response box with nine buttons (DirectIN High-Speed Button, Empirisoft). In the discrimination task, participants used the index fingers of both hands to respond with the extreme left and extreme right buttons. In the reproduction task, they used the right index finger and the extreme right button.

The experiment was divided into 16 short blocks of 32 trials each. Discrimination and reproduction tasks alternated from one block to another, and their order was counterbalanced between participants. A written cue presented for 2 s instructed and reminded participants about the task before each block started. The word “JULGAMENTO” preceded the discrimination task (“judgement” in Portuguese) and “REPRODUÇÃO” preceded the reproduction task (“reproduction” in Portuguese). For both tasks, trials consisted of two visual stimuli (filled circles of 1 visual-degree radius) presented sequentially on a gray screen.

Before each trial started, there was a blank screen (gray background) with a white fixation point at the center (1/6 visual-degree radius) that could last between 900 and 1,200 ms. The first stimulus (S1) was a light blue-filled circle that could last one of four possible logarithmically spaced intervals: 750, 1,042, 1,442, or 2,000 ms. The four intervals occurred equally frequently over the experiment. The order of the intervals presented at S1 was random, with the constraint that a given interval could not occur in more than three consecutive trials. After the first stimulus, another blank screen with a fixation point was shown (600–900 ms). We categorized this segment as the S1-offset epoch.

The second visual stimulus differed according to the task. In the temporal-discrimination blocks, the second stimulus (light green-filled circle) lasted 40% less or 40% more than the first stimulus. After 600 ms from the offset of the second stimulus, a response screen appeared, prompting participants to indicate if the second stimulus was shorter (left button) or longer (right button) than S1. Responses were unspeeded. In the temporal-reproduction blocks, the second stimulus (S2) had the same light-green color, but the participant controlled its duration. Participants were instructed to press the right button when they thought the reference (S1) duration had elapsed. The answer was considered correct if the reproduced duration was longer than half of the reference intervals and less than two times the reference. Feedback was provided for both tasks in every trial. The fixation point turned green if the answer was correct or red otherwise (for 350 ms duration).

Responses exceeding 11.2 s (four times the longest interval for S2 in the discrimination task) were considered a timeout for both tasks. In such cases, no response was registered, negative feedback was provided, and the trial was considered lost. No trials were lost for any participant for reaching the timeout. For the data analysis of the discrimination task, the mean proportion of “longer” responses was calculated separately for trials shorter/longer than the reference and for each reference. For temporal reproduction, trials reproduced more than two times or less than half the presented interval were removed from further behavioral analysis. A linear regression between the presented and produced interval was estimated for each participant.

Before the experiment started, all participants completed a training session consisting of one block of each task. If the participant failed to achieve at least 75% accuracy in either task, the training session would be repeated up to three times. Only two participants required an extra training session.

### EEG recordings and preprocessing

EEG was recorded continuously from 64 ActiCap Electrodes at 1,000 Hz by a QuickAmp amplifier (Brain Products). Data were high-pass filtered online (0.01 Hz) to avoid electrodermal fluctuations. All electrode sites were referenced to FCz and grounded to AFz. The electrodes were positioned according to the International 10-10 system, except for the TP9 and TP10 electrodes in the earlobes. Additional bipolar electrodes recorded the electrooculogram. Data preprocessing was carried out using the FieldTrip toolbox for MATLAB (Oostenveld et al., 2011). Offline filters were applied to the continuous data with a bandpass of 0.1–30 Hz (Butterworth filter, order 3), and all data were re-referenced to the average activity of the earlobe electrodes and downsampled to 250 Hz. Channels with missing data due to problems in acquisition or channels with excessive noise were interpolated using the FieldTrip channel-repair function. Data from most volunteers required no interpolation (14 participants) or up to two channels interpolated. Two participants had three and one participant had four channels interpolated.

Independent component analysis (ICA) was performed for eye movement artifact detection and rejection. Eye-related components were identified with the help of SASICA, available for FieldTrip (Chaumon et al., 2015), by visually inspecting topographies and time series from each component. We used 9 s segments from S1 onset to identify eye movement-related components to be rejected in later analysis-relevant segments. Using these long segments for the ICA, we excluded periods between blocks from the continuous EEG data in which participants could move and interfere with the electoral signal and detection of eye-related components. From this point forward, we used data from 62 channels, excluding bipolar and reference electrodes on the earlobes (TP9 and TP10).

The analyses focused on the processing of the first stimulus. Critically, the first stimulus in both tasks was identical, and participants were not required to make any responses during this phase of the trial. We segmented the data relative to S1 onset (from 150 ms before to 2,700 ms after) and S1 offset (from 150 ms before to 1 s after). Baseline correction used the periods from 150 ms before reference stimulus onset for S1-onset epochs and 50 ms before to 50 ms after offset for S1-offset epochs. Baseline correction for S1-offset epochs was calculated around the 100 ms (−50 to 50 ms) from reference stimulus offset to remove any potential contribution from the classification related to the preceding final segment before offset. We evaluated interval and task decoding during three critical periods: from 0 to 750 ms after S1 onset (since this was the shortest possible S1 duration and therefore present for all trials); at the mean of the last 100 ms before S1 offset; and from 0 to 600 ms after S1 offset (since this was the shortest interval following S1; Fig. 1*b*). Trials were rejected from the analysis if segments exceeded 150 μV or the amplitude range was >250 μV in any of the 62 channels. The same segmentation for S1 and the final 100 ms was used for rejecting noisy trials. The percentage of rejected trials was 2.3% (range between 0 and 11.1%) for S1-onset segments (or the last 100 ms segments) and 0.7% for S1-offset segments (0–5.9%).

### Multivariate pattern analysis

We investigated differences between tasks and intervals in the time-resolved EEG using a supervised learning classification approach. We used a linear discriminant analysis (LDA) as implemented in the MVPA-Light toolbox for MatLab (Treder, 2020) combined with custom scripts. The multiclass classification was performed using the MVPA-Light package. Regularized multiclass was based on LDA. The training data is first projected onto a (C-1)-dimensional discriminative subspace, where C is the number of classes. A test sample is assigned to the class with the closest centroid in this subspace. Decoding performance was estimated using accuracy. For ease of interpretation, classification accuracies were subtracted from chance levels: 0.5 for the two-label task classification and 0.25 for the four-label interval classification.

For the time-resolved classification, data from individual trials were smoothed using a moving average with a 40 ms window. We used a 4-fold cross-validation for task classification (reproduction or discrimination). Each fold consisted of four blocks, two of each task. For example, in the first fold, the test data consisted of blocks 1–4 (two blocks of each task), and the training data consisted of the remaining blocks (5–16, six blocks of each task). In the second fold, test data consisted of blocks 5–8 and so on successively. A 4-fold cross-validation was also used for interval classification within a task, each with two blocks of the task. For example, in the first fold, the test data consisted of the first two blocks of a given task, and the training data consisted of the other 6 blocks; in the second fold, the test data consisted of blocks 3–4 of that task and so on successively. Lastly, we also performed a between-task classification for intervals, with training data consisting of 6 blocks of a task and the test data consisting of the other two blocks of the other task, following the same logic as the within-task classification. Cross-validation is not necessarily needed for the between-task classification, given that the training and test data come from different tasks and blocks. However, we kept the cross-validation so that a single model trained on the same data was tested on the same or the other task. This makes results more comparable within and between tasks.

At each time point, all 62 EEG sensors were used as features. The analysis was conducted at each time point (4 ms apart after downsampling). We evaluated whether accuracy was above chance levels using a permutation test-based control of the family-wise error rate for multiple comparisons (Groppe et al., 2011). This method guarantees a strong control of family-wise error rate (the same degree of false discovery control as Bonferroni’s correction) but is generally more powerful. Permutation-based strong control of family-wise error rate is one of the best methods to establish reliable lower bounds on the onset and offset of effects when a priori boundaries are unavailable (Groppe et al., 2011). In short, permutations are constructed by flipping the sign of a random set of participants and calculating new *t* scores under this rearrangement. For each permutation (*n* = 10,000), a *t*_max_ score is calculated (the most extreme positive or negative value of the *t* scores across all time points) and stored. Critical *t* values and *p* values are estimated from these *t*_max_ distributions. For each test, we report the *t*_max_ value (the estimated *t* value for *p* = 0.05). All tests were one-sided *t* tests compared with zero with an alpha level of 5% for significance level. For time point-by-point classification accuracy measures, we considered time points in which the *t* values exceeded the empirical critical *t* calculated in the permutation test. Whenever relevant, we show univariate *F* values for differences between experimental conditions to illustrate effects.

## Results

### Behavioral results

Participants performed well on both tasks. Using the feedback levels as a measure of accuracy, the mean accuracy for the discrimination task was 91.6%, ranging between 77.3 and 98.0%. The mean accuracy for the reproduction task was 95.7%, ranging between 84.8 and 100%.

For the following behavioral analyses, statistics were calculated using JASP (2022). In the discrimination task (Fig. 1*c*), the proportion of longer responses was submitted to a 2 (shorter/longer than reference) × 4 (S1 duration: 750/1,042/1,442/2,000 ms) repeated-measures ANOVA. There was a main effect of shorter/longer, with participants responding “longer” more often when S2 was longer than S1, as expected (*F*_(1,28)_ = 1,845.78; *p* < 0.001; ω^2^ = 0.98). Additionally, there was a higher proportion for “longer” responses for longer durations of S1 (*F*_(1.741,48.736)_ = 59.90; *p* < 0.001; *ω*^2^ = 0.51; Greenhouse–Geisser sphericity correction). Finally, we also found a significant interaction between these factors (*F*_(2.091,58,536)_ = 14.77; *p* < 0.001; *ω*^2^ = 0.18; Greenhouse–Geisser sphericity correction), indicating that the effect of duration on bias was stronger on both extreme intervals.

For the reproduction task (Fig. 1*d*), participants systematically produced longer responses as S1 duration increased. However, they tended to underestimate longer durations and overestimate shorter ones. The linear regression between the sample and reproduced intervals shows positive slopes (0.71 ± 0.03 mean ± SEM; one-sample *t* test against zero: *t*_(28)_ = 24.8, *p* < 0.001). Despite performing the task well, the pattern of participants’ responses followed the central tendency effect (Jazayeri and Shadlen, 2010).

We compared performance between tasks using two different approaches. In a first analysis, we investigated whether participants with a higher accuracy in discrimination also performed better in the reproduction task, as measured by the root-mean-square error (RMSE). The RMSE measures how far the interval reproductions are from the presented interval, with larger values indicating worse performance. Participants who performed better in one task were also better in the other task. We used a robust skipped correlation to protect against bivariate outliers (Pernet et al., 2013) and observed a significant negative correlation between discrimination accuracy and reproduction RMSE (robust skipped Spearman correlation; *r* = −0.60; 95% confidence interval: −0.79 to −0.29; *p* < 0.001; Fig. 1*e*). Using Spearman's correlation yielded equivalent results (Spearman correlation; *r* = −0.60; 95% CI: −0.79 to −0.33; *p* < 0.001).

In a second analysis, we investigated whether participants were consistent in under-/overestimating the duration of S1 between the two tasks. We correlated the average “longer” responses in the discrimination task and the bias in the reproduction task (calculated as the average difference between reproduced and reference intervals). There was a significant negative correlation between these measures, indicating that participants who reproduced intervals as longer tended to judge the second interval as being “shorter” in the discrimination task (robust skipped Spearman correlation; *r* = −0.42; 95% CI: −0.66 to −0.09; *p* = 0.02; Fig. 1*f*). Using Spearman's correlation yielded equivalent results (Spearman correlation; *r* = −0.42; 95% CI: −0.68 to −0.08; *p* = 0.02).

### Electrophysiological results

#### Timing task modulates EEG signals during S1

**Figure 2.**
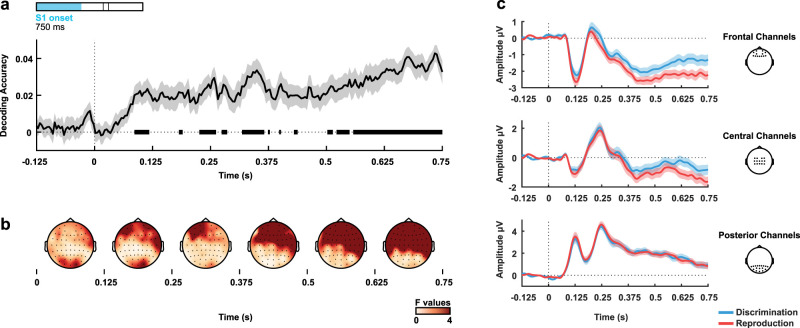
Task decoding during S1. ***a***, Task decoding accuracy above chance level (zero is chance level) throughout the first 750 ms from S1 onset (all valid trials). Bold horizontal lines indicate values significantly greater than zero (*p* < 0.05) from the permutation analysis. ***b***, Univariate *F* values in 125 ms steps from 0 to 750 ms from stimulus onset. Differences between tasks were accentuated in frontal sensors at late periods. ***c***, Grand averages of the signal for the different tasks at frontal (Fp1, AF7, Fp2, AF8, F5, F3, F1, Fz, F2, F4, F6, AF4, AF3), central (FC3, FC1, FC2, FC4, C4, C2, Cz, C1, C3, CP3, CP1, CPz, CP2, CP4), and posterior channels (P8, P6, P4, P2, Pz, P1, P3, P5, P7, PO10, PO8, PO4, POz, PO3, PO7, PO9, O1, Oz, O2). The shaded areas represent the standard error of the mean.

The first analysis focused on the differences in the EEG signal between tasks. We analyzed the first 750 ms from S1 onset from all nonrejected trials. Figure 2*a* shows that the accuracy for classifying the task was statistically above chance level, with effects starting during the early stages of the reference intervals (critical *t* = 3.527). For illustrative purposes, we performed a mass univariate ANOVA with the factors task (discrimination and reproduction) and interval (four possible reference intervals) for each time point and channel. The topographies for the univariate *F* values across sensors in Figure 2*b* shows that differences in the time-resolved EEG between tasks were captured by more frontal and central sensors, as shown in Figure 2*c*. We did not expect any modulation according to the interval during this initial period of S1 (since differential intervals had not yet elapsed), so this analysis also served as a sanity check and there were no significant effects of interval durations (Extended Data Fig. 2-1).

**Figure 3.**
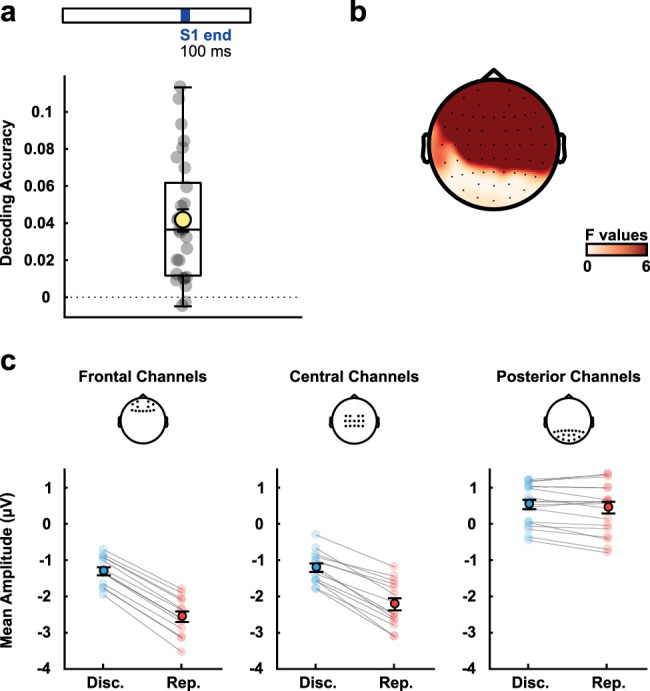
Task decoding in the last 100 ms of the reference stimulus. ***a***, Decoding accuracy above chance level (zero is chance level). The yellow circle depicts the mean accuracy for all participants and the smaller gray circles individual accuracies. ***b***, Univariate *F* values for task differences during the period. ***c***, Mean amplitude of the signal for the different tasks at frontal, central, and posterior channels (using the same channels as in Fig. 2). The light-colored circles depict each participant's values, whereas dark-colored circles depict mean results for all subjects. Error bars represent the standard error of the mean. To visualize activity of all intervals from S1 onset to 600 ms after S1 offset, see Extended Data Figure 3-1.

**Figure 4.**
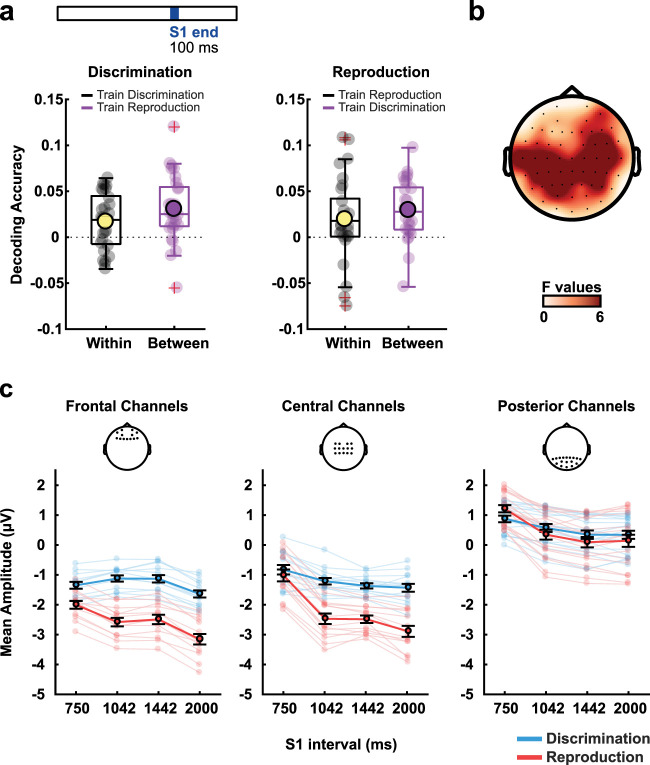
Time-interval decoding in the last 100 ms of the reference stimulus. ***a***, Decoding accuracy for the duration within and between discrimination and reproduction tasks. The yellow and dark purple circles depict the mean accuracies for all participants, and the gray or light purple smaller circles represent individual accuracies. ***b***, Univariate *F* values for interval differences during the period. ***c***, Mean signal amplitude for the different intervals and tasks at frontal, central, and posterior channels (using the same channels as in Figs. 2, 3). For ***a*** and ***c***, the light-colored circles depict each participant's values, whereas the dark-colored circles depict mean results for all participants. Error bars represent the standard error of the mean.

**Figure 5.**
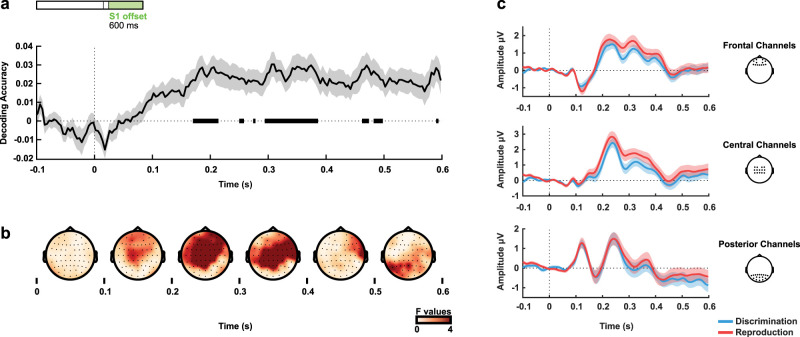
Task decoding after S1 offset. ***a***, Task decoding accuracy above chance level (zero is chance level) after S1 offset. Bold horizontal lines indicate values significantly greater than zero (*p* < 0.05) from the permutation analysis. ***b***, Univariate *F* values in 125 ms windows from 0 to 600 ms from stimulus offset. Task-related differences were more pronounced at central electrodes. ***c***, Grand averages of the signal for the different tasks at frontal, central, and posterior electrodes. The shaded areas represent the standard error of the mean.

**Figure 6.**
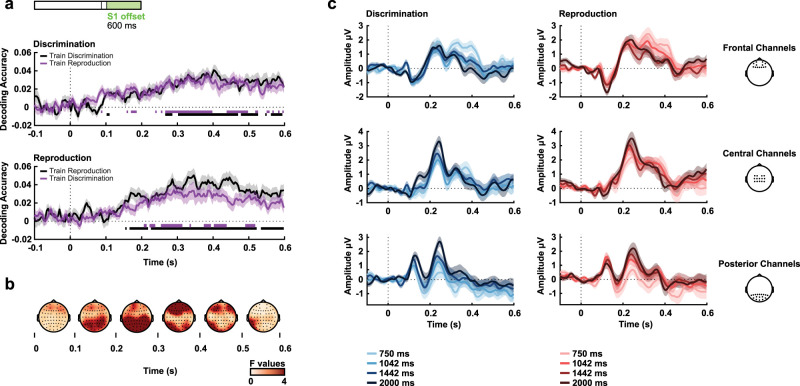
Time-interval decoding after S1 offset. ***a***, Decoding accuracy above chance level in the segment after S1 offset. Bold horizontal lines indicate values significantly greater than zero (*p* < 0.05) from the permutation analysis. ***b***, Average univariate *F* values in 100 ms from 0 to 600 ms from stimulus offset. Differences related to duration were more pronounced at posterior electrodes at early periods (200–300 ms) and frontal at late periods (300–500 ms). ***c***, Grand signal averages for the different durations and tasks at frontal, central, and posterior electrodes (same channel separation as in Figs. 2–5). The shaded areas represent the standard error of the mean.

10.1523/ENEURO.0351-23.2023.f2-1 Figure 2-1 Time-interval classification during S1. As a sanity test, we used the same classifier as used in the other classifications to decode the target duration during S1. Given that the target duration is unavailable to the participant until the end of the interval, we expected to have null results in the decoding accuracy, small F-values for the univariate analysis, and similar ERPs across different intervals. (a) Decoding accuracy during S1 onset, throughout the first 750 ms from S1 onset (all valid trials) (b) Average univariate F-values in 125 ms from 0 to 750 ms from stimulus onset. (c) Grand signal averages for the different time intervals and tasks at frontal, central, and posterior electrodes (same channel separation as in Figures 2-6). The shaded areas represent the standard error of the mean. Download Figure 2-1, TIF file.

#### Task and interval modulate activity at the end of the S1

Information about the duration of the interval is available at the end reference interval. We performed two separate analyses to test whether it was still possible to decode the task and, additionally, decode the duration of the reference interval. Both analyses used the average signal during the last 100 ms before S1 offset for each electrode as the dependent variable.

Classifying tasks yielded a significant above-chance decoding accuracy (*t*_(28)_ = 6.887; *p* < 0.001; Fig. 3*a*). Differences associated with the task were stronger in frontal and central sensors, as shown by the univariate *F* values in Figure 3*b* and *c*.

10.1523/ENEURO.0351-23.2023.f3-1 Figure 3-1 Complete time series of EEG signal grand averages, from S1 onset to 600 ms after S1 offset. Blue (red) indicates discrimination (reproduction) tasks. Grand signal averages for the different time intervals (darker colours indicate longer intervals) and tasks at frontal, central, and posterior electrodes (same channel separation as in Figures 2-6 from the main text). The shaded areas represent the standard error of the mean. Download Figure 3-1, TIF file.

We used both within- and between-task classification approaches to look at interval classification. In the within-task classification, training and testing data came from the same task (i.e., discrimination or reproduction). In the between-task classification, training used data from one task, and testing used data from the other. Significant decoding accuracy values resulted in all cases (Fig. 4*a*). Decoding was significant within tasks for discrimination (*t*_(28)_ = 3.019; *p* < 0.01) and reproduction (*t*_(28)_ = 2.377; *p* = 0.024). Between tasks, decoding was significant when training on reproduction and testing on discrimination (*t*_(28)_ = 4.596; *p* < 0.001) as well as when training on discrimination and testing on reproduction (*t*_(28)_ = 5.025; *p* < 0.001). In contrast to classifying the task, information about durations was most pronounced in central-parietal sensors (Fig. 4*b*,*c*).

#### Task and interval modulate activity after S1 offset

In a second analysis, we investigated whether information about task and interval could also be found in the EEG signal after S1 offset. Only at the offset do participants have complete information about S1 duration. During this period, we found that EEG activity is modulated by both task and duration.

The MVPA classifier decoded which task participants performed early after S1 offset (Fig. 5*a*; critical *t* = 3.500). Importantly, the data for this analysis were baseline corrected around the S1 offset, eliminating the effect reported for the last 100 ms of S1 task classification. Figure 5, *b* and *c*, shows the evolution of EEG differences driven by task as illustrated by univariate *F* values and the average signal at distinct electrodes.

Decoding of intervals was done within and between tasks. Above-chance decoding levels were observed in both (Fig. 6). For the within-task classification, decoding was significant within tasks for discrimination (critical *t* = 3.588) and reproduction (critical *t* = 3.493). Between tasks, decoding was significant when training on reproduction and testing on discrimination (critical *t* = 3.613) and when training on discrimination and testing on reproduction (critical *t* = 3.625). Interestingly, looking at Figure 6*b* and *c*, two patterns emerge concerning the coding of intervals. An early posterior effect (100–300 ms) in which the amplitude is lower for shorter intervals and a later frontal effect (>300 ms) in which shorter intervals show a higher amplitude.

#### Task-dependent time coding after S1 offset

Our final analysis investigated whether intervals are differently encoded between tasks. So far, we have shown that both task and time modulate EEG activity. Our previous analyses, in which time was trained and tested in different tasks, emphasized the commonalities for interval coding between tasks. In this last analysis, we investigated whether revealing selective, task-dependent processing of temporal intervals was also possible.

Our strategy was to use the difference in amplitude from the longest to the shortest interval and test whether MVPA can distinguish whether these differences come from performing different tasks. To increase the number of trials for the classifier, we created pseudotrials comprising these differences. For each pseudotrial, we draw four trials from the 2,000 ms interval (longest) and four from the 750 ms interval (shortest) without replacement. We averaged the signal for each interval and the difference between the longest and shortest signal. Pseudotrials were created using the same 4-fold cross-validation strategy, so training and test pseudotrials came from different folds. In the final step, a similar task classification was used on the pseudotrials, investigating whether the classifier could successfully decode the task from the different subtractions. Creating pseudotrials and classification was repeated 100 times for each participant, and the average accuracy was estimated.

**Figure 7.**
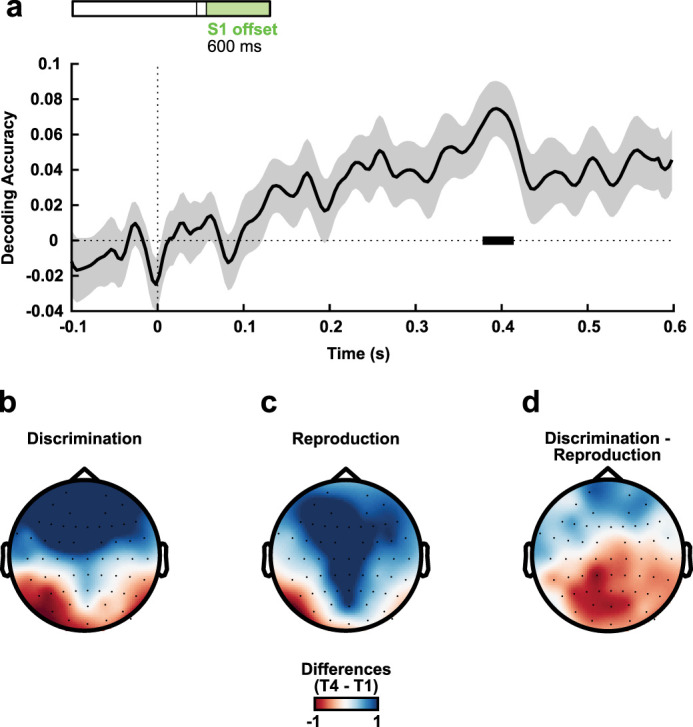
Task decoding after S1 offset on pseudotrials isolating differences between shortest and longest references. ***a***, Decoding accuracy above chance level in the segment after S1 offset. Bold horizontal lines indicate values significantly greater than zero (*p* < 0.05) from the permutation analysis. ***b***, ***c***, Mean differences from the longest to the shortest interval in discrimination and reproduction tasks, respectively, in moments where accuracy was significantly above chance (from 378 to 414 ms). ***d***, Mean difference between ***b*** and ***c*** topographies. To visualize the subtracted activity in each condition, see Extended Data Figure 7-1.

**Figure 8. eN-NWR-0351-23F8:**
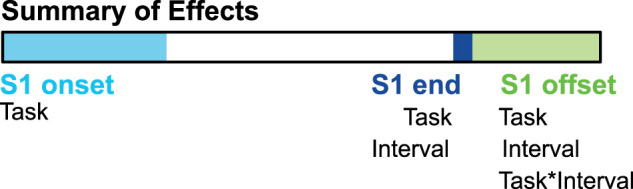
Summary of effects. Schematic representation of the observed effects. We found task differences in brain activity on S1 onset, at the end of S1 and S1 offset. We found differences in brain activity across intervals at the end of S1 and S1 offset. Similar differences were both within and between tasks. Task-dependent differences in temporal intervals were found only after S1 offset.

Task-dependent differences in duration processing appeared after the offset of S1. No differences occurred during the last 100 ms of each interval (mean accuracy of 0.014; standard error of the mean, 0.018; test difference from zero: *t*_(28)_ = 0.747, *p* = 0.461). Differences after S1 offset are shown in Figure 7*a*. The periods in which the MVPA accuracy was above chance (critical *t* = 3.388) overlapped with periods in which we also observed common interval–duration coding between tasks (Fig. 8). Figure 7, *b* (discrimination) and *c* (reproduction), shows the average differences from the longest to the shortest interval for discrimination and reproduction tasks, respectively, when the classifier accuracy was significantly above chance (from 378 to 414 ms). Figure 7*d* depicts the difference between discrimination and reproduction topographies (discrimination − reproduction). [Fig eN-NWR-0351-23F8]

10.1523/ENEURO.0351-23.2023.f7-1 Figure 7-1 Grand averages of the EEG signals differences for extreme intervals (interval 2000 ms - interval 750 ms). Difference between the evoked activity by the longest and shortest interval for both tasks in frontal, central, and posterior electrodes (same channel separation as in Figures 2-6). The shaded areas represent the standard error of the mean. Download Figure 7-1, TIF file.

## Discussion

Using MVPA, we revealed a set of overlapping neural signals for encoding the duration of temporal intervals for explicit perceptual judgment and motor reproduction. The different task demands also activated task-specific processes across various time scales, both during the interval itself and after its offset, revealing possible parallel states of motor versus perceptual preparation. Lastly, we also found evidence that task demands can interact with duration estimation. Despite analyzing brain activity during periods with identical stimulation and timing demands, neural signals discriminating between the future task demands of perceptual judgment versus motor reproduction were also prevalent across the various time scales analyzed, ranging from early during the encoding interval to after the offset of the reference interval.

Task-specific EEG activity at the early stages of interval processing differed between encoding durations for subsequent perception- or action-based tasks, suggesting that timing unfolds in different neural contexts depending on future task demands. Within these different contexts, not only was it possible to decode timing within each task, but decoding generalized between tasks, suggesting a common activity pattern that covaried with time. This common encoding of time was observed during both periods in which timing information was available—at the end of the reference interval and after its offset. After the interval offset, the decoding of intervals was significant from early periods, including early differences over posterior sensors and later effects distributed over frontal sensors.

There has been an increase in studies that use MVPA to investigate EEG activity. Using such methods has several advantages, as they can naturally account for and leverage the multivariate nature of the signal (Michel and Murray, 2012; Stokes et al., 2015) while also effectively addressing the statistical challenge of multiple comparisons. In our study, we capitalized on these methods as a data-driven approach to look for possible differences between tasks and at similar covariations of neural activity as a function of time across them. This brings essential advances, given that previous studies have focused on looking for differences rather than possible similarities when comparing activity across tasks. However, we have also examined the underlying event-related potentials across conditions to compare our findings with previous results.

For the interval onset period, differences between tasks were driven by an early activity and followed by what resembles a contingent negative variation (CNV) in frontal sensors, with a stronger CNV in the reproduction task. One possibility for the observed difference between tasks is that the reproduction task is more difficult than the comparison task. However, previous studies that have looked at the CNV during the encoding of an interval found differences in the CNV even when tasks had similar performance (Schlichting et al., 2020) or displayed no CNV differences when one task was easier than another (as found in Bueno and Cravo, 2021). Another possibility is that the motor component in the reproduction task might play a role in modulating the CNV, given the intrinsic relationship between time and motor regions (De Kock et al., 2021). These results strengthen the possibility that the brain can tap into a common repertoire of processes to different extents depending on the task demand. Future studies combining a wider range of temporal and nontemporal tasks with different motor demands will be essential to explore these relationships further. Lastly, the term CNV has been used for a wide variety of different brain activities and tasks. In explicit timing tasks, it often refers to EEG activity during the presentation of the target duration, which participants have to judge as shorter/longer than a reference (Pouthas et al., 2000; Macar and Vidal, 2003; Gontier et al., 2009; Ng et al., 2011; Kononowicz and van Rijn, 2014; Bueno et al., 2017). Studies that have looked at CNV-like activity during the encoding of an interval are less frequent and present mixed findings, with some studies finding more negative CNV for timing tasks compared with number tasks (Schlichting et al., 2020). In contrast, others do not find CNV differences for tasks in which participants encode time or color (Kulashekhar et al., 2016; Bueno and Cravo, 2021).

For the offset period, we also found both task decoding and time decoding that generalized across tasks. Specifically, we found a consistent duration decoding starting at ∼200 ms post offset for reproduction and 300 ms for discrimination. The early activity was more present in posterior sensors, resembling a P2, while the late activity was more concentrated in central sensors, resembling a P300 or a late positive component (LPC). There has been an increase in studies that have looked at post-offset EEG activity in timing tasks. These studies have focused on early post-offset activity (Kononowicz and van Rijn, 2014) and, more recently, on the LPC (Wiener and Thompson, 2015; Bannier et al., 2019; Baykan and Shi, 2022; Ofir and Landau, 2022; Baykan et al., 2023; Özoğlu and Thomaschke, 2023). As with the CNV, the term LPC has been used for various post-offset activities that vary in sensors and periods, making it hard to pinpoint whether different studies capture activity from similar processing stages. Critically, and similar to the CNV, most studies have looked at the LPC during the target interval presentation and not during its encoding.

To our knowledge, only three other studies have looked at post-offset activity during interval encoding (Bueno and Cravo, 2021; Damsma et al., 2021; Kruijne et al., 2021). In our previous study (Bueno and Cravo, 2021), a similar pattern, although smaller, was found only for the later activity starting at ∼300 ms after the interval offset in frontal sensors. EEG correlates with duration were also reported after the offset of auditory-marked durations (Damsma et al., 2021). In this case, effects also resembled a P2 with larger amplitudes for longer intervals, similar to our parietal P2. Finally, Kruijne and colleagues did not find duration effects on either P2 or LPC, although they focused their analysis on central sensors while using visual targets (Kruijne et al., 2021). The different topographic distributions of the results suggest that the earliest effects after offsetting a reference stimulus include sensory processing involving central sensors for the auditory task and posterior sensors for our visual task. Future studies could test for systematic variations in early timing-related signals depending on the sensory modality of the reference and test intervals.

Complementing our observation of robust cross-task decoding of temporal intervals, we also discovered that task demands impacted duration estimation. These task-dependent temporal encoding effects became apparent only after the offset of the reference interval. Unlike the common temporal encoding effects, the selective processes were not observed toward the end of the reference interval. The reason is unclear. Part of the explanation may rest on the need to derive the differences between stimulus durations between trials, which may have reduced the sensitivity and power of our analysis. Alternatively, the stimulus offset may be necessary to increase the readout of neural differences by acting as a perturbation that makes manifest differences in states that may otherwise remain encoded in “activity-silent” patterns of synaptic weights (Wolff et al., 2015, 2017). Moreover, after the interval offset, participants may start transforming and gating the encoded interval information to guide performance in the specific upcoming task. Such a process is likely to include the interaction between timing signals and proactive engagement of sensory versus motor systems for perceptual judgment versus motor reproduction, respectively. The differential encoding and maintenance of temporal intervals depending on how timing signals will be used are consistent with recent views of how working memory is flexibly coded depending on future use (Nobre and Stokes, 2019). Theoretical (Orhan and Ma, 2019) and empirical results in nonhuman primates (Warden and Miller, 2010) and fMRI in humans (Muhle-Karbe et al., 2017) have found that how information is maintained may depend on the expected use.

The simultaneous occurrence of an interaction, indicating selective timing-related computations between tasks, and cross-generalization, indicating common timing-related computations across tasks, can seem contradictory if one adopts a simple view that brain processing only unfolds sequentially through one single circuit. Our finding, instead, suggests that timing-related brain processes can unfold in tandem through different circuits. In this pluralistic scenario, some timing operations may be shared and lead to a significant degree of (though not absolute) cross-generalization. In contrast, other timing operations may be local and selective, leading to a significant degree of (though not absolute) pattern divergence.

There has been an increasing interest in hybrid models for temporal processing that propose a combination of local task-dependent areas interacting with partially distributed timing mechanisms (Merchant et al., 2013). Our findings align with the hybrid model proposal. We found a correlation in performance across tasks in our behavioral results and similar duration-dependent activity across tasks in our EEG results, suggesting common processing. However, we also found evidence for task-specific activity and an interaction of task-dependent activity and durations. These differences do not seem to be driven by tasks not being equally challenging, given that we found good performance in both and that we used short and intercalated task blocks to prevent learning or fatigue from modulating one task more than the other. However, we cannot completely rule out the possibility that general difficulty or motivation for the different tasks modulated EEG activity. On the other hand, the observed task-specific encoding of time shows how even these factors could modulate timing specifically.

In conclusion, our findings provide evidence that sensory versus motor demands may influence time encoding. These differences can have implications for other proposed categorizations of temporal processes, such as implicit versus explicit tasks. To gain a more comprehensive understanding of the neural mechanisms involved in temporal processing, future studies could employ higher spatial and spectral resolution techniques in humans and nonhuman animals to investigate these dimensions more systematically. In particular, modulations of activity in different frequency bands have been noted in timing studies (Sperduti et al., 2011; Kulashekhar et al., 2016; Wiener et al., 2018; Grabot et al., 2019; Kononowicz et al., 2020; Schlichting et al., 2020), but a coherent picture is yet to emerge. Systematic differences in frequency modulation according to a task's perceptual and motor demands (van Ede et al., 2020) would indicate engagement of specialized, local temporal processing. A combination of spectral analysis and multivariate approaches should provide a fruitful approach and yield valuable insights into the complex interplay of cognitive and neural factors that underlie our perception of time.

## Synthesis

Reviewing Editor: James Howard, Brandeis University Decisions are customarily a result of the Reviewing Editor and the peer reviewers coming together and discussing their recommendations until a consensus is reached. When revisions are invited, a fact-based synthesis statement explaining their decision and outlining what is needed to prepare a revision will be listed below. The following reviewer(s) agreed to reveal their identity: Martin Wiener, Assaf Breska. Note: If this manuscript was transferred from JNeurosci and a decision was made to accept the manuscript without peer review, a brief statement to this effect will instead be what is listed below.

Thank you for submitting a timely and thorough revision of your manuscript. Overall the reviewers feel that you have addressed their main points. Some minor issues regarding description of statistical and analysis methods remain to be addressed

1. There remains a lack of sufficient detail in the description of some of the behavioral analyses. Please describe which ANOVA model was used in the analysis of the discrimination task (Fig. 1c), and provide inferential statistics for the regression analysis of reproduction (Fig. 1d).

2. Please indicate exactly how the tmax was calculated in your permutation-based control of FWER. Was this value calculated for each time point of the decoding analysis individually, or was this calculated as the most extreme positive or negative t-value across all time points?
