# Supplementary figures and images for: Time for What? Dissociating Explicit Timing Tasks through Electrophysiological Signatures
Source: eNeuro. 2024 Feb 21;11(2):ENEURO.0351-23.2023. doi: 10.1523/ENEURO.0351-23.2023 (PMC10884563; doi:10.1523/ENEURO.0351-23.2023)

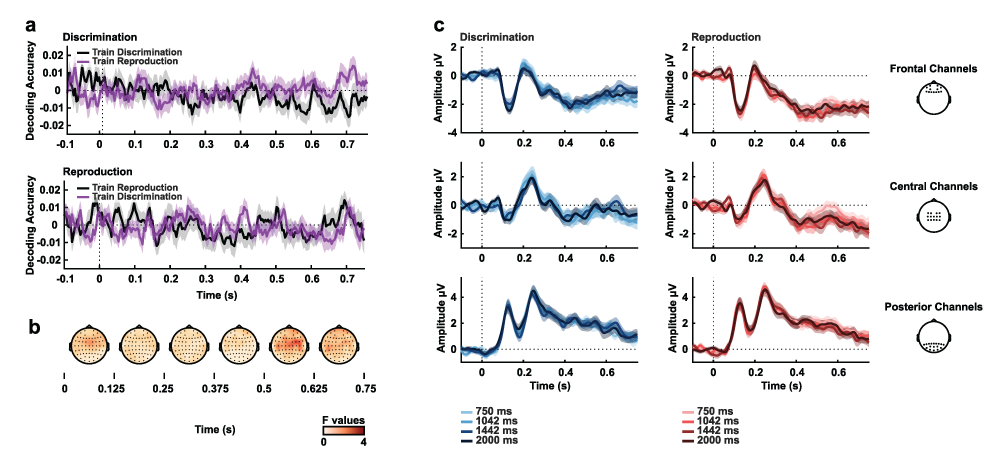

Supplement: Figure 2-1 — Time-interval classification during S1. As a sanity test, we used the same classifier as used in the other classifications to decode the target duration during S1. Given that the target duration is unavailable to the participant until the end of the interval, we expected to have null results in the decoding accuracy, small F-values for the univariate analysis, and similar ERPs across different intervals. (a) Decoding accuracy during S1 onset, throughout the first 750 ms from S1 onset (all valid trials) (b) Average univariate F-values in 125 ms from 0 to 750 ms from stimulus onset. (c) Grand signal averages for the different time intervals and tasks at frontal, central, and posterior electrodes (same channel separation as in Figures 2-6). The shaded areas represent the standard error of the mean. Download Figure 2-1, TIF file. [file eneuro-11-ENEURO.0351-23.2023-s001.tif]

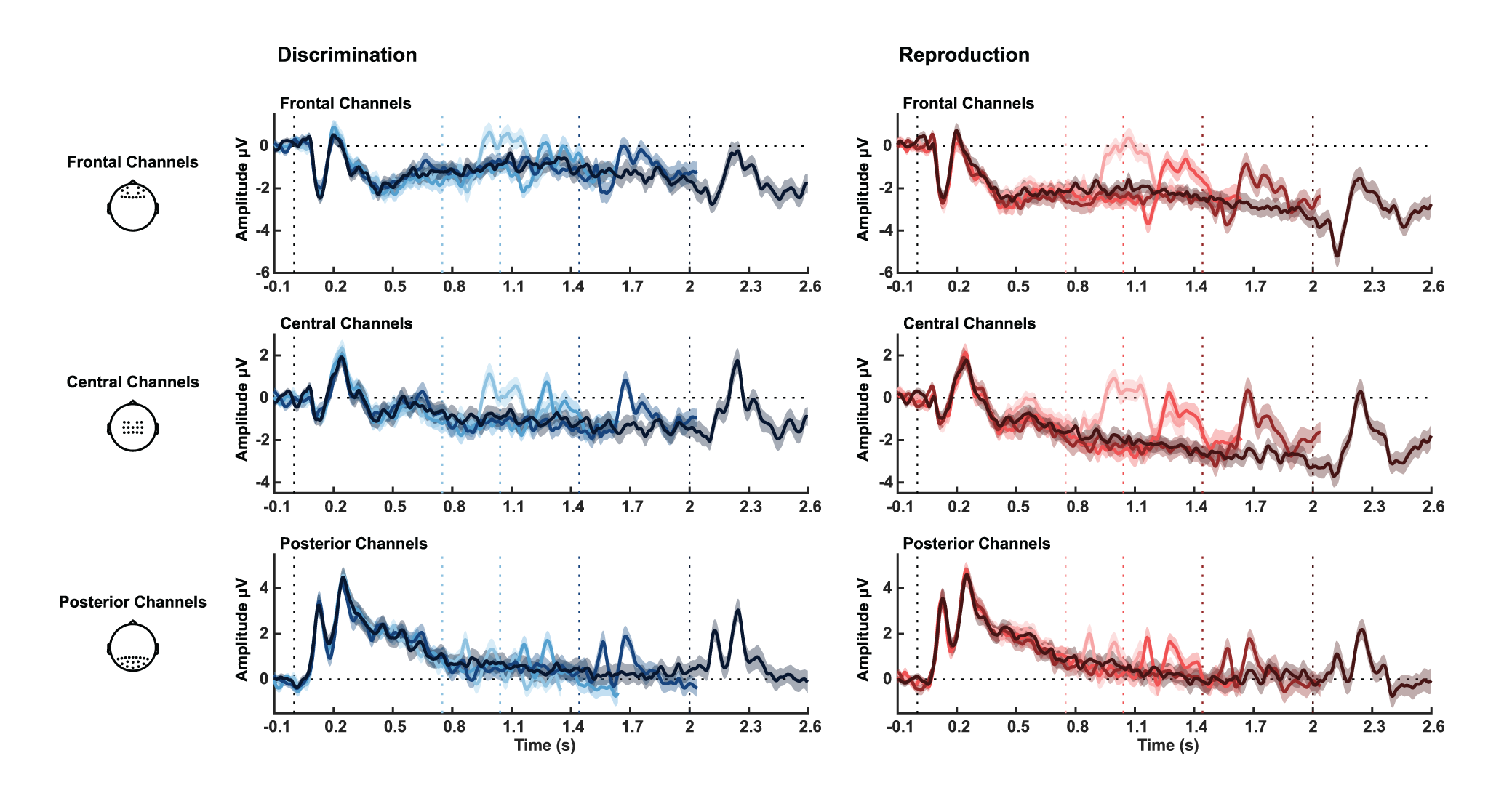

Supplement: Figure 3-1 — Complete time series of EEG signal grand averages, from S1 onset to 600 ms after S1 offset. Blue (red) indicates discrimination (reproduction) tasks. Grand signal averages for the different time intervals (darker colours indicate longer intervals) and tasks at frontal, central, and posterior electrodes (same channel separation as in Figures 2-6 from the main text). The shaded areas represent the standard error of the mean. Download Figure 3-1, TIF file. [file eneuro-11-ENEURO.0351-23.2023-s002.tif]

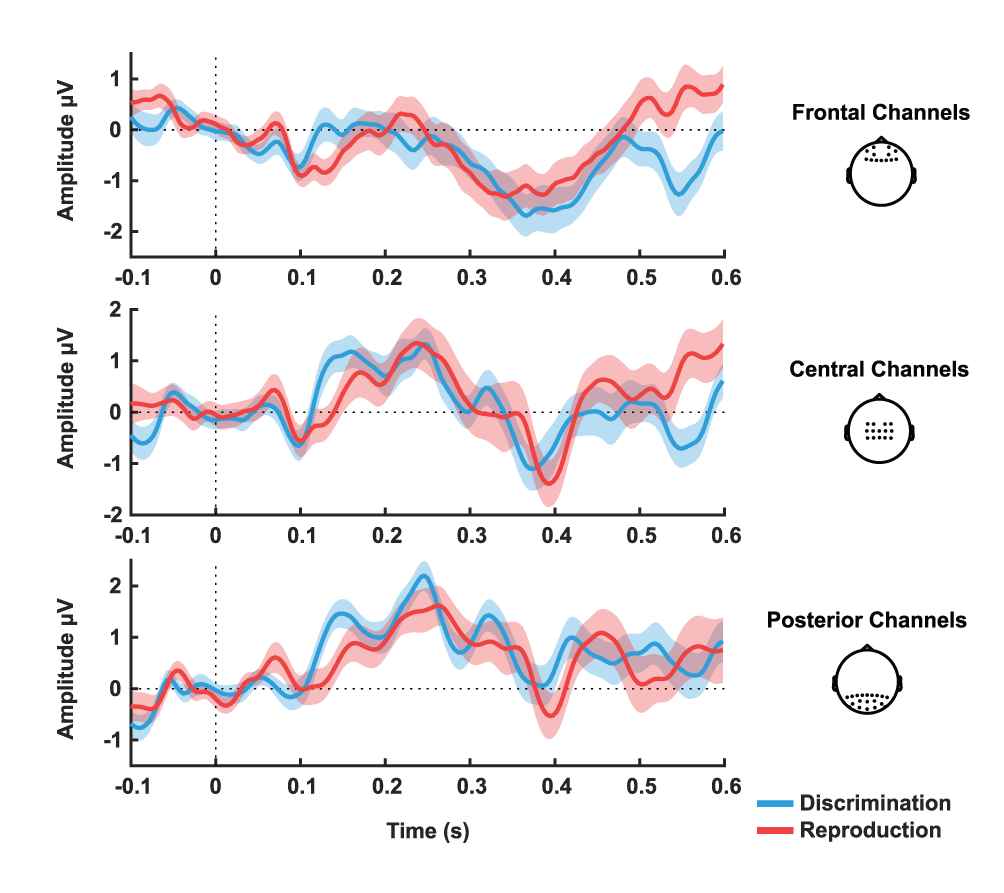

Supplement: Figure 7-1 — Grand averages of the EEG signals differences for extreme intervals (interval 2000 ms - interval 750 ms). Difference between the evoked activity by the longest and shortest interval for both tasks in frontal, central, and posterior electrodes (same channel separation as in Figures 2-6). The shaded areas represent the standard error of the mean. Download Figure 7-1, TIF file. [file eneuro-11-ENEURO.0351-23.2023-s003.tif]
